# Stereotactic Ablative Radiotherapy versus Lobectomy for Operable Stage I Non-small-cell Lung Cancer: A Pooled Analysis of Two Randomized Trials——胸外科医生的解读

**DOI:** 10.3779/j.issn.1009-3419.2015.06.12

**Published:** 2015-06-20

**Authors:** 林 许

**Affiliations:** 210009 南京，江苏省肿瘤医院胸外科 Department of Toracic Surgery, Jiangsu Tumor Hospital, Nanjing 210009, China

最近美国MD Anderson癌症中心胸部肿瘤放疗科的张玉蛟教授在国际著名杂志*The Lancet Oncology*上发表了一项随机研究结果^[1]^。该研究汇总分析了2项（共58例）对比立体定向消融放疗（stereotactic ablative radiotherapy, SABR）和手术在可手术Ⅰ期非小细胞肺癌（non-small cell lung cancer, NSCLC）作用的随机研究。该研究对可手术的Ⅰ期NSCLC患者应用SABR和目前标准的治疗方法（有创性手术治疗）的总生存期、复发率及毒性进行了对比分析，研究结果表明手术组及SABR组的3年生存率分别为79%和95%，3年无复发生存率分别为80%和86%；手术组中有6例患者死亡，而SABR组中仅1例死亡；SABR组未发现严重毒副反应。

这项研究的发表注定会成为全世界肺癌研究领域的焦点和热点。众所周知，外科手术治疗可手术的早期NSCLC已经有一个世纪之久，疗效肯定，安全可靠；相对而言，SABR作为一种较为新颖的无创性治疗手段，虽经多个中心、多篇文献证实其同样有效，但其在过去的十余年间大多用于治疗不能耐受手术或不可手术的NSCLC患者。在肺癌治疗领域这两种治疗手段孰优孰劣还缺乏真正意义上的临床随机对照研究结果进行权威评判。

作为一名肺癌外科医生，笔者在认真拜读了这篇可能改变现有早期NSCLC治疗模式的文章后，谈一谈个人对该研究的感想，以期抛砖引玉，得到更多肺癌工作者的回应。

首先，正如作者在文中所提到的，该研究最大的局限性即入组样本的数量以及随访时间的不足。该研究两组累计仅入组58例患者，对于一项有可能改变现今临床经典治疗模式的前瞻性、随机对照研究而言，这样的样本量明显不足，因此在这样小样本研究对象的基础上得到的研究结果很有可能会发生误差和偏移；另外我们都知道，肿瘤患者的经典随访时间通常为1年、2年和5年，该研究仅仅比较3年的总生存率和无复发生存率显然也是不够的。而且由于随访时间较短，对于SABR治疗可能的远期毒副反应，甚至放疗导致的辐射致癌等问题亦有可能疏漏。

其次，该研究入组患者的年龄结构问题也是许多肺癌外科医生热议的话题之一。我们注意到在该研究中手术组患者最长者为85岁。众所周知，虽然高龄并非肺癌手术的绝对禁忌，但高龄患者肺叶切除术围手术期并发症发生率和死亡率均较高，肺癌外科医生对于75岁以上的高龄肺癌患者在手术（特别是非微创的常规剖胸术）前往往会进行非常谨慎的评估。在临床实际工作中恰恰有相当多的高龄Ⅰ期NSCLC患者被外科医生推荐进行SABR治疗。因此我们猜想本研究中手术组较高的死亡病例数、较低的3年生存率是否与入组患者的年龄构成相关。换言之，如果剔除了高龄患者后进行长期随访的话，该项研究可能会得出不同的结果。

再次，我们注意到手术组入组患者的手术方式为肺叶切除术（包括常规开胸和微创）。肺癌的微创外科是近年来普胸外科的热点之一。经过广大肺癌外科同仁的不懈努力，现在肺癌外科微创技术已经相当成熟和稳定，单孔胸腔镜技术、腔镜肺段切除术等更为微创的技术正逐步开展，大大提高了早期肺癌患者的生活质量。因此，我们设想如果该研究中手术组入组患者多为接受微创治疗（胸腔镜下肺叶切除、肺段切除等）的早期肺癌患者，那研究结果有否可能发生改变？

最后，我们不得不从外科的角度强调淋巴结清扫的必要性。我们注意到在该研究中SABR组中有4例患者发生了区域淋巴结的复发转移，病例数和相对比例均高于手术组，这说明临床Ⅰ期NSCLC患者系统性的淋巴结清扫同样重要。这也正是手术治疗优于无创治疗手段的因素之一。

在此需要申明的是以上观点绝非本人试图否定这项研究的结论，虽然在VALOR和SABRTooth等更多的研究结果出来之前还很难定论手术或SABR孰优孰劣，但可以肯定的是手术或SABR均是Ⅰ期NSCLC患者可靠的治疗手段之一，我们需要将每种治疗手段的适用人群进行精确分层，根据患者的具体情况个体化选择，这应该也是现在盛行的“精准医疗”的具体体现吧。
